# Manifestations, social impact, and decay of conceptual beliefs: A cultural perspective

**DOI:** 10.1002/brb3.3470

**Published:** 2024-04-01

**Authors:** Rüdiger J. Seitz, Raymond F. Paloutzian, Hans‐Ferdinand Angel

**Affiliations:** ^1^ Department of Neurology, Centre of Neurology and Neuropsychiatry, LVR‐Klinikum Düsseldorf, Medical Faculty Heinrich Heine University Düsseldorf Düsseldorf Germany; ^2^ Department of Psychology Westmont College Santa Barbara California USA; ^3^ Institute of Catechetic and Pedagogic of Religion Karl Franzens University Graz Graz Austria

**Keywords:** beliefs, believing, credition, coding, home, hope, narratives, politics, rituals, semantic, transcendence

## Abstract

**Introduction:**

Believing comprises multifaceted processes that integrate information from the outside world through meaning‐making processes with personal relevance.

**Methods:**

Qualitative Review of the current literature in social cognitive neuroscience.

**Results:**

Although believing develops rapidly outside an individual's conscious awareness, it results in the formation of beliefs that are stored in memory and play an important role in determining an individual's behavior. Primal beliefs reflect an individual's experience of objects and events, whereas conceptual beliefs are based on narratives that are held in social groups. Conceptual beliefs can be about autobiographical, political, religious, and other aspects of life and may be encouraged by participation in group rituals. We hypothesize that assertions of future gains and rewards that transcend but are inherent in these codices provide incentives to follow the norms and rules of social groups.

**Conclusion:**

The power of conceptual beliefs to provide cultural orientation is likely to fade when circumstances and evidence make it clear that what was asserted no longer applies.

## Graphical abstract

Believing comprises multifaceted processes that integrate information from the outside world through meaning making processes with personal relevance. Conceptual beliefs can be about autobiographical, political, religious, and other aspects of life and may be encouraged by participation in group rituals. Assertions of future gains and rewards that transcend but are inherent in these codices provide incentives to follow the norms and rules of social groups.

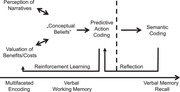

## 1 INTRODUCTION

Humans live in a complex, rapidly changing environment about which they hold various beliefs. Because beliefs are, thus, widely prevalent, an interdisciplinary initiative recently put the current research on beliefs into perspective (https://www.frontiersin.org/research‐topics/23734/credition—an‐interdisciplinary‐approach‐to‐the‐nature‐of‐beliefs‐and‐believing). Accordingly, humans are exposed continuously to information from stationary physical objects as well as from events with a perceivable beginning and end (Asprem & Taves, 2022). The information is processed in formal probabilistic terms and valued with respect to its subjective relevance in a bipolar fashion with effort and cost on one side and benefit and reward on the other (Seitz et al., 2009, 2018). This allows for predictions of future events with respect to possible benefits or costs when it comes to decisions about one's behavior. On the neural level of individual agents, processing of the formal perceptive content takes place in the cerebral cortex, whereas processing of the subjective value involves the subcortical, so‐called reward pathway in the nucleus accumbens in the ventral striatum. In the basal ganglia, incentive‐encoded contextual memories are converted to reward prediction by the action of dopamine and endogenous peptides (Castro & Bruchas, 2019; Depue & Morrone‐Strupinsky, 2005). The implementation of beliefs in the human brain is accounted for by a distributed model of in‐parallel organized cortico‐subcortical networks that integrate discrete and continuous processes (Friston et al., 2017). Thereby, different information maintained in the different cortico‐subcortical circuits can also be brought into register across functional modalities.

Notably, the sensory information about objects and events is processed rapidly in a probabilistic prelinguistic bottom–up fashion without conscious awareness and is stored in memory for later retrieval if and when needed (Seitz et al., 2023a, 2023b). People intuitively trust their perceptions because they are processed with ease and are typically accurate representations of the environmental stimuli (Brashier & Marsh, 2020). These probabilistic and emotionally loaded representations reflect an individual's experience, including what people believe intuitively about their social relations. It has recently been proposed that based on the properties of the information, they can be categorized as primal beliefs, as summarized in Table 1 (Seitz & Angel, 2020). From a neuroscience perspective, trusting in what has been perceived and is believed is learned by outcome prediction and has been found to involve the medial frontal cortex for confirmatory evidence and the lateral prefrontal cortex for alternative outcomes (Akaishi et al., 2016).

**TABLE 1:** Input‐based belief classification.

Input from environment	Objects	Events	Narratives
Categorical level	Empirical beliefs	Relational beliefs	Conceptual beliefs
Descriptive level	Primal beliefs		Thematic subtypes

*Note*: Beliefs have been categorized with respect to the type of environmental information they reflect (Seitz & Angel, 2020). Beliefs about objects and events that manifest instantaneously without dependence on language have been labeled descriptively primal beliefs. Conceptual beliefs are based on narratives and comprise different subtypes, including autobiographic, political, and religious themes.

Humans are unique in their ability to generate, understand, and respond to language as we know it. Because of this ability, they can process symbolic information from narratives in various forms from infancy onward, including via nursery rhymes and fairy tales (Nelson, 2003). Moreover, children are told how to behave properly as well as what is right and what is wrong. Later, they may be exposed to other narratives such as those about the history and faith of their family and the family's traditions and ritual behaviors (Table 1). On the basis of narratives transmitted in their social environment, humans can construct conceptual beliefs about themselves, their ancestors, their belonging to a faith community, and the probability that they will achieve personally meaningful goals in the short‐ and long‐term future. Recently, it was proposed that it is the linguistic character of information that separates conceptual from primal beliefs (Seitz & Angel, 2020). As humans also become able to read texts in newspapers, books, or in the present era on digital devices, they are in the position to read or hear descriptions of life in previous times, the experiences of other people, and of other parts of the world. That humans have these abilities supports the notion that the evolutionary foundations of social life are cooperation within groups and compliance with social norms in culturally marked groups (Claessens et al., 2020).

In this article, we employ a social cognitive neuroscience approach (Lieberman, 2007) to describe the manifestation, social impact, and decay of conceptual beliefs. We review recent research on how believing in culturally important concepts can be differentiated into beliefs about various themes, including autobiographical accounts of the self, political and related issues, and matters pertaining to religion or spirituality. We clarify that conceptual beliefs are expressed like primal beliefs, in a first‐person perspective. But they do not directly address someone's personal experience of the physical environment because they pertain to social life, which nevertheless may also include reference to the physical environment in which social life takes place. In addition, we explain that conceptual beliefs may include goals to attain, ideas about benefits or costs, or ideas for how to achieve a certain state in the future. Thereby, they typically translate into decisions about behavior and may have implications that transcend the status quo. We argue that although conceptual beliefs may be maintained over extended periods of time, they may nevertheless change based upon new personal insights or scientific findings. Ultimately, the third‐person inference inherent in the above description of conceptual beliefs will lead us to explain the meta‐analytic nature of talking about beliefs.

## 2 BELIEVING IN CULTURALLY IMPORTANT CONCEPTS

**FIGURE 1:**
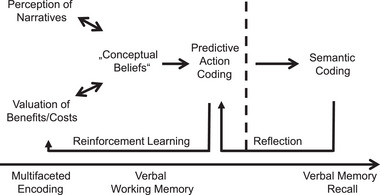
Comprehensive model of the role of conceptual beliefs for coding of human behavior. The model is based on the integration of environmental information with personal relevance resulting in representations in the human brain that can be labeled “conceptual beliefs.” The double‐headed arrows signify bottom–up and top–down processing. The model accounts for the storage of conceptual representations in memory and for their modifications by prediction errors via reinforcement learning as well as for modification of the generated behavior by explicit reasoning. Broken line signifies the transition to conscious awareness. *Source*: Adapted and further developed from Seitz et al. (2022, 2023b).

Because people learn from infancy onward that their sensory perceptions typically reflect the properties of their physical environment in a reliable manner, they are used to believing them (Brashier & Marsh, 2020). Similarly, people get exposed to narratives from infancy onward often in the ritualized context of festivals that are entertained in families and faith groups. Thereby, the inherent concepts acquire a meaning to them. According to the comprehensive model of believing, narratives may become personally relevant for an individual and determine his or her actions by predictive coding (Figure 1). Additionally, people may become aware of what they believe and thus remember it and express it verbally as propositions. These capabilities open up the possibility that a person can reflect upon what he or she believes and how to act accordingly. Even so, it should be kept clear that believing is mediated by neural processes in the human brain, whereas beliefs are the results of these processes and are stored as stable representations in the brain. This implies that in common language as well as in scientific discussions we should be clear to distinguish between beliefs and the processes of believing. Unfortunately, all too often the distinction between them is not made—belief being a noun (i.e., a thing) and believing being a verb (i.e., an activity). As outlined recently, believing is modulated by new information that either concurs or contradicts an individual's predictions that were based on hitherto held beliefs (Seitz et al., 2023a). This is the essence of the credition model (Angel, 2022). Accordingly, the processes that mediate believing in concepts can be differentiated from the beliefs themselves on linguistic, physiological, and semantic grounds (Table 2).

**TABLE 2:** Comparison of believing concepts and conceptual beliefs.

Believing	Belief
Linguistic level	
Verb	Noun
Processing mode of narratives	Categorical label for classified contents
Physiological level	
Neuropsychic function	Processing result
Below awareness	Involvement of awareness
Perceptive action and emotional appraisal	Inferential explanation with subjective meaning
First‐person perspective	Third‐person attribution
Fluid modification	Stable representation
Subjective preferences	Objective classification
Semantic level	
Probabilistic intuition	Idealized construct
Neural embodiment	Meta‐analytic description

*Note*: There are different levels to describe beliefs. On each level there are analogues characterizing believing.

To further clarify and add some detail, conceptual beliefs provide people with predictive frameworks for how to navigate their social and cultural environments. They can be differentiated into three thematic subtypes: The first subtype concerns autobiographical information on which people base their self‐esteem, perception of their own skills and abilities, and so on. The second subtype includes beliefs about public affairs, political concerns, people's positions and constraints within organizations, and roles that people may fill in a social system. Especially important in this thematic subtype, the beliefs also include concepts about morality and norms for how to behave in a social system, that is, how to distinguish right from wrong without compromising the group's values or negatively affecting other people (Shermer, 2016). The third subtype includes narratives about religious or spiritual issues. They provide frameworks for humans to believe that their lives and behavior matter and are meaningful, that is, that they matter in some transcendent sense—whether earthly or other‐worldly. Let us now unpack the meanings that underpin these three thematic subtypes of conceptual beliefs.

### 2.1 Autobiographical beliefs

Autobiographical beliefs result from narratives and cultural myths stored in autobiographical memory (Nelson, 2003; Fivush et al. 2011). This unique human long‐term memory system of the self emerges from a number of specialized subfunctions mediated by cortico‐subcortical neural circuits (Markowitsch, 2013). People rely on the autobiographical narratives they learned during childhood and adolescence. Typically, these narratives are about who they are, their ancestors, and where they belong, including which country and perhaps even which continent. All these social identity processes are central to how a person develops individual autobiographical memory (Abrams & Hogg, 2010; Hogg et al., 2017). Thus, humans develop beliefs about their physical environment and the members of the social groups to which they belong (Scoboria et al., 2004). As recently highlighted, such beliefs include highly emotionally laden concepts, such as home, hometown, and homeland (Wei et al., 2023). Another important aspect of such beliefs is that they offer or imply hopes about the future that transcend the reality of people's lives in the here and now (Oviedo, 2022). For example, in the Western patriarchal societies of history, children were declared legitimate descendants and therefore were entitled to bear the name of the family and inherit their parent's property. A testable hypothesis would be that autobiographic narratives have social and cultural impact when hierarchical structures evolve in a given population.

### 2.2 Beliefs about political and public affairs

Beliefs about political and public affairs play a constitutive role in the social environment as they promote in‐group cohesion and intergroup aversion (Claessens, 2020). Thereby, concepts about morality and norms are of importance because they provide rules for how to behave in a socially accepted and viable manner. In addition to being defended by individual ego‐ and group‐justifying motives, beliefs about politics are also used to justify policies and procedures at a system level (Jost et al., 2022). The latter is termed system justification and is defined as the system‐serving tendency to defend, bolster, and legitimize aspects of the societal status quo. Moreover, evaluating someone's social status includes integrating perceptual cues from the person's behavior with knowledge‐based information about the past and present circumstances (Mattan et al., 2017). Thus, because beliefs about political matters may include explicit or implied visions of a future highly desired or feared by those who believe them, they can be held very strongly and be resistant to change. As a consequence, inflexible oppositional stances may result from discussions with people who hold opposing political beliefs. For example, liberals and conservatives—a distinction that refers to Western tradition—were shown to differ in that liberals endorse individualizing moral foundations, whereas conservatives primarily endorse binding foundations (Stewart & Morris, 2021). But the liberals and conservatives did not differ in their physiological responses to threat, which implies that these two groups hold their (oppositional) moral foundations with relatively equal strength (Bakker et al., 2020).

### 2.3 Beliefs about religion and spirituality

People want to understand their environments and how they change because they would otherwise have to live with constant uncertainty about what is going to happen. Beliefs within certain religions have been found to help decrease such uncertainty by providing concepts and teachings, and sometimes prophecies, about what will happen and how it can be expected to occur (Hogg et al., 2010; Teehan, 2006). Such concepts about supernatural agents, unseen gods, and other divinities may become highly believable and therefore influential for religious individuals (Plante et al., 2023; van Elk, 2022). Today, beliefs about religious issues are understood to be similar to other beliefs and rooted in the same neuropsychological capacities. That is, they have a biological basis and may be linked to the cerebral operations that take place in the individual human mind/brain (Ernandes, 2018). Moreover, such beliefs and promises about future events and associated norms are functional because they help people decide what do to, guide their behavior, and may facilitate choosing wisely in difficult situations. In particular, recent evidence from cross‐cultural studies shows that models of ethics, morality, or striving for God's favor can be beneficial to individuals and local communities (Purzycki et al., 2017). Partly countering this idea, however, Sapolsky (2017, p. 621) has endeavored to balance the scales by arguing that religion can be seen as *an incredibly powerful catalyst for both our best and worst*. Even so, although across certain religions people believe in an afterlife and pray, the frequency of prayer, types of prayer performed, and estimates of religiosity vary among religions and in different countries (McCleary & Barro, 2006).

Beliefs rooted in religions entertain important social and cultural roles, as they may promote or prevent social cooperation between groups and among different individuals within a group. Beyond that, they may also regulate conflict between groups and interpersonal competition (Norenzayan et al., 2016) as well as promote conflict and terrorism (Juergensmeyer, 2017). Moreover, individuals’ reputations built upon beliefs about relational or social issues may help sustain cooperative relationships among unrelated individuals in groups and other social systems (Romano et al., 2021). Relatedly, moral judgments typically based on narratives held in groups and societies have been found to develop in response to observed violations of moral norms (Malle, 2021). It has recently been hypothesized that the dynamics of dissonance between moral and social beliefs predict subsequent belief change (Dalege & van der Does, 2022). However, beliefs about social interactions and beliefs about morality are known to operate on different levels of awareness (Seitz et al., 2023a). Thus emerges the interesting question of whether it is external information or an individual's internal attitude that governs the stability of a belief versus the change of one's belief in favor of another. A researchable hypothesis is that the development of values in an individual is rooted in the interaction among empirical, relational, and conceptual believing processes.

## 3 BELIEVING IN THE FUTURE TRANSCENDING THE PRESENT

Let us now extend the above discussion and describe how whatever people are experiencing may intuitively build up conceptual beliefs with transcendent meanings for the future. People experience themselves instantaneously in the here and now. But when paying attention to other people, someone's mind may tend to transcend its own experiences to include situations and settings in a more remote future time or place, whether only imagined or genuinely believed to be forthcoming (Liberman & Trope, 2008). This process is likely to be mediated by predictive coding that guides how people plan for near and distant situations (Figure 1). Accordingly, humans are inclined to develop views, hopes, and beliefs about personal and societal affairs that transcend the present circumstances (Mesulam, 2008). The capacity to transcend and to sense transcendence (be it understood as world‐immanent or as world‐transcending) reflects the aptitude to elevate something people have performed from the individual momentary level of relevance to the level of general relevance and timelessness. In fact, a sense of self‐transcendence is a typical result of having attributed meaning to what one has done (Sugiura, 2022) and thus implies social acknowledgement in a horizontal dimension by contemporary mates and in a vertical dimension by successors and children. This is expressed verbally, for example, as “the good will win,” “the truth will show up,” “work first, then distraction is ok,” “I respect my responsibility,” “I will not lose myself,” or “I do my duties.” Even so, beliefs about self‐transcendence are personal and, thus, do not necessarily have a meaning to anyone else.

Narratives found in religions often function to enhance the sense of transcendence. We highlight that the adjective “religious” is predominantly used in relation to the notion of religion rather than to religiosity or religiousness (Angel, 2022). This is probably because the notion of religion has changed profoundly over the course of history. For example, in Ancient Roman times religion was somewhat of a juridical term, whereas in Christianity it was understood as an attitude of faith and belief (Angel, 2020). Moreover, the Western term *religion* is not adequate to be used in relation to Buddhism or Hinduism, although it is commonly applied to them in Western terminology. Although space constraints for this article prevent a deeper comparison of the great religions, it may be sufficient to mention that commandments and narratives in the Bible, which includes both the so‐called Old Testament with the holy scriptures of Judaism and the Christian New Testament, make claims about the future. For instance, they include the anticipation of a long and safe life with respect by others, an eternal life that transcends this earthly life, and a final judgment. They also warn prophetically about catastrophies resulting from false attitudes or wrong behavior. However, the language in which such notions are communicated may be important. For example, the English term *promise* has two German translations (Versprechen, Verheißung). Promise in a Christian sense (Verheißung) transcends the notion of Versprechen. In an analogous way, in Buddhism, it is said that a person who does not cling to earthly possessions but does engage in meditation will achieve full insight and have internal peace. In an Islamic tradition, a martyr is believed to be rewarded with virgins in the afterlife. Hindus believe in reincarnation, although a human in this life may be reincarnated as a member of a different species. Typically, these promises are believed by their followers and are part of the glue of faith communities. From history, we know that they can also motivate their members to undertake huge common projects such as building overwhelmingly large structures, such as temples, churches, and shrines. In addition, they may induce people to participate in heroic or dangerous activities, such as crusades, pilgrimages, and wars. Importantly, such phenomena are not limited to religious faith communities.

Participation in and traditional practice of rituals held in common may promote a sense of transcendence and be perceived and sensed by each participant as a feeling of greatness (Gelfand et al., 2020). Moreover, humans may tend to make attributions about supernatural agents, which is a widespread practice throughout the world (Exline & Wilt, 2022). Rituals enhance the feeling of being related to other individuals and can facilitate the sense of transcendence shared among groups of people. For example, a baptism, wedding, or funeral service may become highly emotionally loaded with the implication of an act of singularity for the persons involved that is conceived to be of relevance beyond the individuals who are present. Notably, listening to and performing music, including chanting, plays an important role for the acquisition of rituals. This is particularly the case in adolescents whose psychomotor development is highly moldable by brain plasticity (Alcorta & Sosis, 2005). Feelings of transcendence may be perceived as matters with global implications and for eternity. Thus, psychologically, a sense of transcendence can develop its meaning in the fullest way only in the social context of a faith community, whether it be religious, political, familial, ethnic, or other (Hogg et al., 2010). Such development often begins in childhood and is integral to the formation of the feeling of belonging to a certain group such as a family, community, and traditionally a nation. The feeling may be exaggerated and develop into a feeling of being special, such as belonging to an elect group or one with a special mission. To illustrate, in many traditional societies of the past and in some societies of today, women have been said to be protected against unauthorized assaults and abuse by being required to conform to clothing guidelines (shadors, long suits, etc.) and by restrictive rules of behavior (living within the family until marriage, not walking out of the house alone, and especially not in the dark). And in virtually all cultures, weddings have been established as extraordinary events that, among other things, signify the legitimacy of the couple's children. Conversely, agnostic members of modern, pluralistic societies may sometimes give the impression that in present‐day weddings and funerals, something that touches them is missing, as highlighted by the German philosopher Jürgen Habermas (Boman & Rehg, 2017).

## 4 FADING OF CONCEPTUAL BELIEFS IN SOCIAL CONCEPTS

Even given the above argument about the acquisition, pervasiveness, and importance of conceptual beliefs, we must also clarify that it is possible for the strength to which the beliefs were held and conveyed meaning to individuals and groups to diminish over time. Members of religious groups are known to share norms, rules about what is moral and immoral, and expectations that most of them may have learned during childhood. For example, in Western countries, teachings within Christianity about a blessed future may have been so powerful that some people chose to adhere to the norms and rules even if they had to accept monetary losses and other deprivations. And perhaps more powerful than that, some believers seem to have been driven by a different motive grounded in what they understood as a deeper truth, for example, doing works of charity. Other religions include comparable conceptual beliefs reflected in their teachings and recommendations for how to live and be in relationships with others. Nevertheless, a person's conceptual beliefs may change over his or her lifetime because as people age, they may attribute different meanings to the same words or concepts (Knäuper et al., 2016). Conceptual beliefs of the kind described here, including promises of a future state of affairs, can be expected to lose their credibility as soon as competing claims are made by other faith groups and are appraised as more salient (Sharot et al., 2023). Due to a reduced strength of such beliefs and an associated loss of social coherence, faith communities may be in danger of dissolving. This has been happening with increased frequency in Christian churches in Western Europe and North America in recent years. Nevertheless, the development of an interior life or practice of prayer may still be serving as psychological “capital” for increased resilience and coping in, for example, the Christian‐orthodox refugees from the war in Ukraine (Oviedo et al., 2022). Meanwhile, believing that the future will provide a world in which all people live together in peace or that humans will succeed in reconciling a strong economy and a sustainable ecology seems to weaken. In addition, contrary beliefs in the benefit or harm of the development of artificial intelligence are apparent.

## 5 SEMANTIC CODING OF CONCEPTUAL BELIEFS

Figure 1 schematically illustrates that people may become consciously aware of what they believe. They can thereby express what they believe from a first‐person perspective in statements starting with “I believe …” (Oakley & Halligan, 2017). Empirical evidence suggests that people use “believe” preferentially in religious or political contexts but say “think” in confidence statements about facts (Heiphetz et al., 2021). This distinction corresponds to the difference between the affective involvement in believing in contrast to the more emotionally detached aspects of what one thinks about facts (van Leeuwen et al., 2021). Statements about beliefs may predominantly be dichotomic, because someone can either share a belief with another person (and state “I believe that …”) or disagree with the other person (and state “I do not believe that …”). The dichotomic character of belief statements appears to manifest because humans’ sensory systems and experiences of their environment are similar, so that their first‐person descriptions of their environment are also similar. Analogously, the probabilistic nature of conceptual beliefs corresponds to the implied or actual character of their claims or ideas about the future in a graded fashion because they are matters of degree, even though they may be expressed in categorical, binary terms (Dietrich & List, 2018). Importantly, when a person says: “I know” or “I know that …,” they are saying something very different from “I believe that ….” The expression “I know” does not relate to abstract knowledge, as illustrated in algebraic expressions (e.g., 3 + 5 = 8). The expression “I know” appears to reflect a person's momentary subjective conscious awareness. The probability of error in such statements may increase over time, however, because of the longer time interval between encoding and retrieval of information from memory. In accord with the notion that consciousness is related to the magnitude of information processing in the brain (Greenfield & Collins, 2005), it has been shown that awareness occurs when the amount information reaches a threshold of detectability and becomes available for active processing by the individual (Sonnberger et al., 2023).

Semantic coding transfers the contents of what one believes from the level of unattended processing to the level of reflection and reappraisal in first‐person perspective (Seitz et al., 2022). Narratives can be memorized and recited later without having to constantly pay attention to them. Thus, they are “learned by heart.” However, verbally expressing beliefs makes it possible to convey their contents to others directly during conversations or indirectly via written texts—which enables communication of their contents to other people in other places and times. It is important to realize, however, that semantic coding of conceptual beliefs includes many degrees of freedom. In the most extreme case, a person may believe P but may report non‐P for a deceptive purpose (Feierman & Oviedo, 2020). Or the person may have understood someone else's belief only partially and therefore may convey it incorrectly. In particular, various aspects of conceptual beliefs are known to differ among different families and social groups. Nevertheless, both intuitive and deliberate behavior can implicitly express what someone believes. For example, how people wash their hands has been shown to reduce the perceived discrepancy between preferred and rejected alternatives in decision‐making (Lee & Schwarz, 2010).

Typically, people who hear or read verbal statements are in a position to reflect on them. Someone could be emotionally touched by a statement and feel positive about it, remain neutral, or find it annoying or offensive. Moreover, bystanders may evaluate someone's stated belief and behavior and conclude that the person acted in a proper way or failed to do what is right and in conformity with his or her purported ethics. In essence, humans can modulate their behavior when aware of their options and do not necessarily have to be slaves of their emotions. Some may prefer to behave altruistically because they feel they are guided by promises in which they believe. When people in Western societies communicate with others about what they believe, they tend to use nouns for concepts such as knowledge, belief, mind, culture, economy, and God, analogous to how they use nouns for objects, such as tree, table, and book. However, a noun has to be defined, and when people talk to each other they need to be sure that they are talking about the same thing. They need to agree on the definitions of the words they use, because only this can guarantee that they understand each other. This complexity explains why the interpersonal exchange of conceptual statements can be slower than verbal exchange about objects or events.

Finally, we emphasize that talking about conceptual beliefs is a post hoc attribution in third‐person perspective to an inferred state in an individual that becomes apparent after hearing what the person says and observing his or her behavior. Table 2 illustrates the correspondence of the cognitive neuroscience processes affording believing concepts and the resulting conceptual beliefs at the linguistic, physiological, and semantic levels. More broadly, beliefs act as fundamental hypotheses about the world in the presence of sensory and environmental uncertainty (Fritsch et al., 2023; Oeberst & Imhoff, 2023) Consequently, beliefs do not appear to be propositions expressed by consciously aware, so‐called rational agents, but rather have to be considered as determinants of people's spontaneous and intuitive behavior in a complex world. We argue that this also pertains to conceptual beliefs as outlined in this article.

## 6 DISCUSSION

The novel achievement promoted by a cognitive neuroscience perspective is that believing is a central, multifaceted brain function that is composed of a number of fundamental psychological processes such as perception and valuation of external information, memory encoding, predictive coding, and decision‐making (Seitz et al., 2023a). The composite processing of language‐bound information determines the resulting conceptual beliefs (Figure 1). Believing puts individuals in the position to act adequately in the momentary environmental context. Importantly, however, a person's beliefs are not accessible directly by another individual but may only be inferred from a person's verbal or overt behavior. In contrast to primal beliefs, conceptual beliefs are specifically human products of brain activity in the form of ideas such as emotionally laden concepts derived from narratives (Table 1). New insights in the light of the pertinent literature suggest the following.

### 6.1 Comprehensive model of belief formation

In accordance with Brashier and Marsh (2020), we argue that humans, like nonhuman primates, are disposed to believe that their senses provide an accurate and reliable representation of the objects and events in their environment. Believing is likely to play an important role in the consistency of an individual's actions by supporting the development of preferences (Vogt, 2022). In this respect, humans are similar to nonhuman primates (Maravita & Iriki, 2004). Our comprehensive model of primal beliefs goes beyond the cybernetic input–output model that was popular 50 years ago, according to which perceptual processing includes a predefined response generated according to the structure‐determines‐function principle (Key et al., 2022). Instead, our model accounts for additional processes of valuation in terms of possible rewards, costs, and punishment that are known to be mediated via the ventral striatum (Castro & Bruchas, 2019; Schultz, 2016). Moreover, it is consistent with the principle of predictive coding for selection of behavior that is mediated in parallel cortico‐subcortical loops in accordance with the free‐energy principle (Friston, 2010; Friston et al., 2017). In particular, this neuroscience‐based model accounts for the integration of sensory perception of external information with automatic or intuitive meaning making (Park, 2022, 2010). The result is that the multilevel orchestration of believing enables organisms to successfully navigate their physical and social environments (Sugiura et al., 2015). Importantly, believing is brought about by neural processes that afford subjective preferences and behavioral decisions in a first‐person, ex ante perspective. These processes do not require conscious awareness or linguistic functions and pertain to narratives as constitutes of conceptual beliefs in a comparable fashion (Seitz et al., 2023a).

### 6.2 Social consequences of conceptual beliefs

Conceptual beliefs can include personal reports and fairy tales as well as stories about people's past and explanations of their present circumstances. Their meanings in terms of content and emotional loading differ among individuals, because each person has a different history (Cyrulnik, 2005). Ancient versions of such narratives often included statements about what to expect in the future. When such statements are understood as promises, they are likely to be believed by the followers and provide some justification for holding the belief due to the inherent potential reward. As noted above, for example, Christians believe in the final judgment and a future life in heaven after death, and Muslims believe that they will spend eternity with Allah in the hereafter. These examples illustrate how people can believe in concepts that extend into the future for nonevidence‐based reasons (Longheed & Simpson, 2017). People can hold religious or political beliefs without any explicit awareness of their structure in a manner similar to how they can use language without metacognitive awareness of its grammatical rules (Claessens et al., 2020).

Carrying our argument further to social cognitive neuroscience (Lieberman, 2007), the key to solidarity and cooperation in heterogeneous communities is to extend prosociality beyond close social networks and in‐group boundaries to include unknown and dissimilar others (Baldassarri & Abascal, 2020). It has been said that prosocial behavior is based on five clusters of emotions (van Kleef & Lelieveld, 2022). These emotions are associated with opportunity and affiliation; they include happiness, contentment, hope, and appreciation as well as self‐transcendence, including gratitude, awe, elevation, and compassion. In contrast, emotions and related states that work against intergroup harmony include distress, supplication, sadness, disappointment, fear, anxiety, dominance, and status assertions, including anger, disgust, contempt, envy, and pride. Lack of appeasement and social repair for wrongs done that lead to feelings of guilt, regret, shame, or embarrassment have also been identified as important factors. Similarly, supraordinate concepts such as love and peace can be disrupted when there is a discrepancy of predictions between primal and conceptual beliefs resulting in aversive behavioral states, such as distrust, jealousy, and hate (Seitz, 2024). Thus, two important aspects of the evolutionary foundations of social life are those emotional and related states that support cooperation within groups and across wider interdependent networks, as well as conformity to norms of morality and concepts within cultures and smaller groups (Claessens et al., 2020, Teehan, 2016). Norms of morality are to be obeyed, while offending them can lead to punishment. Relevant to our present times, it has been proposed that believing fake news is associated with uncareful reasoning and a lack of relevant knowledge about what is believed, combined with acceptance of what is being communicated without careful thought based on simplistic heuristics such as familiarity with and liking of the source of the communication (Pennycock & Rand, 2021). Dysfunctional behavior of this sort has been found to be augmented by a gap between what people believe and what they share on social media. In this regard, there seems to be a need in the social science of societies to state explicitly what the moral foundations of public communications are (Zaman, 2021).

### 6.3 Conceptual beliefs and cognitive neuroscience

We have outlined in this article that the results of the processes of believing are conceptual beliefs descriptively classifiable according to the content to which they refer (Table 1). Although what these semantic labels mean in a specific case is unclear, they all have a familiar sound and are generally understood (e.g., autobiographical beliefs, beliefs about the economy, religious beliefs, beliefs about political and social affairs, etc.). In contrast, the expression “normative belief” is ill‐defined. The most clearly spoken norms in Christianity are the Ten Commandments. They are statements of the form: “You shall …” and “You shall not ….” Such statements, however, are not about beliefs. They are demands. How demands can become part of conceptual beliefs is not discussed in this article. We highlight only a few aspects: For instance, the statement “I believe it is good to take care for your next door neighbor” reflects a subjective empathic involvement and expresses one's attitude that it is good to take care of others. This is different from the statement “I believe in charity,” which reflects believing in a concept. It is wise to be aware that the colloquial saying “There is good reason to believe that …” is a meta‐cognitive statement from a third person perspective; it conveys that someone may tend to believe something similar to the person whose behavior he or she has observed. In comparison, each Arabic number reflects only one value, which is specific and can be observed directly by counting parts of one's own body. For example, children who are in the process of learning how to add or subtract frequently use their fingers to objectify their calculations. Basic arithmetic calculations are concrete rules that, in principle, can be visualized, explained, and thereby learned. Thus, communication about numbers is straightforward, whereas communicating abstract words and narratives is far more difficult and noisy and may therefore fail.

Conceptual beliefs reflect the “reality” for the believing individuals but have been found to change in content when there are changes in how the beliefs are defined. Such changes in definitions reflect cultural changes in world views that are brought about by, for example, novel technical developments. A major illustration of this is that improved optical instruments were the basis for an irreversible change from a geocentric to a heliocentric world view. Moreover, although Plato established the concepts of a mortal physical body and an ideal immortal soul, Aristotle favored a conceptual approach that focused on human psychological capacities (Bennett, 2007). In recent years, cognitive neuroscience has opened a pathway to an empirical understanding of the “implementation level” of cognitive capacities including perception, valuation, memory encoding, action generation, and decision making, as well as believing (Seitz et al., 2023b). Similarly, as fake news contradicts reality and the concepts associated with certain verbal expressions may become distorted in totalitarian political systems, communication and social relations among individuals are likely to be compromised and eventually may deteriorate entirely in such conditions. Because it has been shown that successful self‐regulation is dependent on top–down control from the prefrontal cortex over subcortical regions involved in reward and the balance of emotion (Heatherton & Wagner, 2011), failure of self‐regulation occurs whenever the balance is skewed by particularly strong impulses in subcortical areas or when prefrontal function is impaired. Thus, there is increasing evidence that psychic functions are, indeed, processed in the brain with a defined topographical distribution of intensively connected areas and temporal determinants (Axer & Amunts, 2022; Fastenrath et al., 2022). Changes such as those noted above affect the way we will consider primal and conceptual beliefs and support the notion that evolved in the European history of philosophy that the mind reflects psychological capacities in a being equipped with language (Bennett, 2007). Consistent with this notion, religious individuals engaged in improvised prayer were shown to have neural activation in the left‐hemispheric language areas, such as the temporopolar region, the anterior medial prefrontal cortex, the temporoparietal junction, and bilaterally the precuneus—all areas that are related to social cognition (Schoedt et al., 2009). It was hypothesized that praying to God is an intersubjective experience comparable to “normal” interpersonal interactions. Because contemporary neurosciences have affected social constructs such as law, state, and market, our discussion also touches on the recently developed concept of privacy, which provides means for responsibility to and for oneself (Grant, 2021).

Ultimately, what a person believes may be thought of as an essentially individual matter (Hacker, 2001). In addition, such beliefs may include attributions about supernatural agents—attributions whose widespread relevance has recently been highlighted (Exline & Wilt, 2022). But people also differ in how they reason about the causes of events and the attributions they subsequently make. From a philosophical point of view, these differences highlight that there is a difference between believing something to be and believing something to be true (Hacker, 2001). How humans process and come to understand narratives is even more complex. It is well known that verbal transmission can be faulty at the sender's and the recipient's end, which leads to difficulty in understanding what has been said. In addition, people may differ in the degree to which what they hear or read is subjectively relevant. Because of this, different individuals may have contradictory attitudes about concepts and beliefs, which can trigger highly emotional and even violent arguments. The points noted above assume that the perceived correspondence between thought and reality is orchestrated within language, not between language and reality (Hacker, 2001). In any case, ritual acts and routines that different people have in common have been shown to help bring them together and foster similar beliefs in individuals that differ as well as within social groups (Gelfand, 2020). Thus, it seems that conceptual beliefs in groups may be strengthened and maintained through activities that people have in common such as learning at school and other group activities. But as the power of conceptual beliefs fades when circumstances and evidence make it clear that what is asserted contradicts reality, the associated norms can also be seen to lose their authority.

## 7 CONCLUSION

The multilevel study of believing and beliefs is an interdisciplinary endeavor. Believing is a central capacity of the brain that evolved in phylogeny. It affords adaptive behavior in the diverse and ever changing and complex world. The manifestation of conceptual beliefs is intimately intertwined with human language and the evolution of normative and moral concepts in human societies. Thereby, conceptual beliefs are fundamental to the evolution of social life and the formation of human culture. Above that, conceptual beliefs can be objects of reflection and, thus, have been in all times topics of philosophy and historical and religious sciences.

## AUTHOR CONTRIBUTIONS

R.J.S designed and drafted the manuscript. R.F.P and H.F.A edited and complemented the text.

## CONFLICT OF INTEREST STATEMENT

Nothing to declare.

## FUNDING INFORMATION

The work was not based on dedicated funds.

### PEER REVIEW

The peer review history for this article is available at https://publons.com/publon/10.1002/brb3.3470.

## Data Availability

This is a theoretical article without experimental data on its own. All references of the data referred to are cited in the text and listed in the References.
